# Recruiting to a Randomized Controlled Trial of a Web-Based Program for People With Type 2 Diabetes and Depression: Lessons Learned at the Intersection of e-Mental Health and Primary Care

**DOI:** 10.2196/12793

**Published:** 2019-05-24

**Authors:** Susan Fletcher, Janine Clarke, Samineh Sanatkar, Peter Baldwin, Jane Gunn, Nick Zwar, Lesley Campbell, Kay Wilhelm, Mark Harris, Helen Lapsley, Dusan Hadzi-Pavlovic, Judy Proudfoot

**Affiliations:** 1 Department of General Practice University of Melbourne Carlton Australia; 2 Black Dog Institute Sydney Australia; 3 School of Psychiatry University of New South Wales Sydney Sydney Australia; 4 School of Medicine University of Woollongong Woollongong Australia; 5 Diabetes and Metabolism Division Garvan Institute of Medical Research Sydney Australia; 6 Centre for Primary Health Care and Equity University of New South Wales Sydney Sydney Australia

**Keywords:** e-mental health, primary care, patient recruitment, depression, type 2 diabetes, learning

## Abstract

**Background:**

E-mental health (eMH) interventions are now widely available and they have the potential to revolutionize the way that health care is delivered. As most health care is currently delivered by primary care, there is enormous potential for eMH interventions to support, or in some cases substitute, services currently delivered face to face in the community setting. However, randomized trials of eMH interventions have tended to recruit participants using online recruitment methods. Consequently, it is difficult to know whether participants who are recruited online differ from those who attend primary care.

**Objective:**

This paper aimed to document the experience of recruiting to an eMH trial through primary care and compare the characteristics of participants recruited through this and other recruitment methods.

**Methods:**

Recruitment to the SpringboarD randomized controlled trial was initially focused on general practices in 2 states of Australia. Over 15 months, we employed a comprehensive approach to engaging practice staff and supporting them to recruit patients, including face-to-face site visits, regular contact via telephone and trial newsletters, and development of a Web-based patient registration portal. Nevertheless, it became apparent that these efforts would not yield the required sample size, and we therefore supplemented recruitment through national online advertising and promoted the study through existing networks. Baseline characteristics of participants recruited to the trial through general practice, online, or other sources were compared using the analysis of variance and chi square tests.

**Results:**

Between November 2015 and October 2017, 780 people enrolled in SpringboarD, of whom 740 provided information on the recruitment source. Of these, only 24 were recruited through general practice, whereas 520 were recruited online and 196 through existing networks. Key barriers to general practice recruitment included perceived mismatch between trial design and diabetes population, prioritization of acute health issues, and disruptions posed by events at the practice and community level. Participants recruited through the 3 different approaches differed in age, gender, employment status, depressive symptoms, and diabetes distress, with online participants being distinguished from those recruited through general practice or other sources. However, most differences reached only a small effect size and are unlikely to be of clinical importance.

**Conclusions:**

Time, labor, and cost-intensive efforts did not translate into successful recruitment through general practice in this instance, with barriers identified at several different levels**.** Online recruitment yielded more participants, who were broadly similar to those recruited via general practice.

## Introduction

The potential for e-mental health (eMH) interventions to address many of the challenges faced by health care systems globally has seen them receive increasing attention from researchers and policy makers alike. eMH interventions provide an opportunity for individuals affected by mild-to-moderate symptoms of mental health disorders to access low-cost evidence-based treatments and aim to reduce the burden on providers while maintaining patients’ connection to the broader health system.

The value of eMH interventions may be particularly pertinent to primary care clinicians who are responsible for the majority of mental health care [1]. However, although the efficacy of eMH interventions is now well established [2], their implementation into routine care remains limited [3].

Of the 13 eMH randomized controlled trials (RCTs) included in a recent meta-analysis [2], only one recruited participants through primary care [4]. Some of this evidence gap is attributed to difficulties in conducting research in this setting, which are by no means unique to trials of eMH interventions. Primary care–based research accounts for a disproportionately small proportion of all health care research [5], and only one-third of RCTs recruit to target [6]. Documented barriers at the patient, practitioner, and organizational levels contribute to these difficulties [7]. As such, many randomized trials of eMH interventions to date have instead sourced participants online [2]. This approach is generally considered effective and efficient, but there is also evidence to the contrary [8-10], and the degree to which these samples are representative of the broader population has been questioned [11,12].

There is a need to correct the dearth of primary care–based research and for greater transparency and reporting of the issues researchers are facing in this setting. Our recent SpringboarD RCT [13,14] provides an opportunity to examine these issues in relation to an eMH intervention for people with mild-to-moderate depressive symptoms and type 2 diabetes (T2D)—2 highly prevalent and commonly comorbid conditions in primary care [15,16].

This paper has described an intensive and ultimately unsuccessful approach to primary care recruitment and the recruitment strategies that were required to supplement it and reports the outcomes of each. It then explored how participants recruited through primary care compare with those recruited through other avenues on key demographic and clinical characteristics. Such data help identify the tensions inherent in primary care that impact on recruitment.

## Methods

### Study Overview

The SpringboarD RCT (trial registration ACTRN12615000931572) [13,14] examined the effectiveness of a Web-based cognitive behavioral therapy–based self-help program (myCompass, Black Dog Institute) for improving work and social functioning and depressive symptoms in people with depressive symptoms and T2D at 3, 6, and 12 months compared with a placebo control program.

Our joint focus on eMH and primary care led us to design our recruitment strategy accordingly. First, we aimed to recruit through general practices in the 2 most populous states of Australia (New South Wales and Victoria). To supplement this approach, we also recruited through national online advertising and promoted the study through existing clinical and research registries. All promotional materials invited interested individuals to visit the SpringboarD website to learn more about this *wellbeing project for people with T2D*. After reading the trial information, interested participants provided informed consent and completed a screening questionnaire to determine eligibility. Those eligible then completed a baseline assessment and indicated how they learned about SpringboarD by selecting a referral source from a drop-down menu. They were then randomized to use the myCompass program or an active placebo control program for 12 weeks. The trial was approved by the University of New South Wales Human Research Ethics Committee (HREC; No. 15090) and registered with the University of Melbourne HREC (No. 1545422).

### Participants

The target sample size was 600 participants at baseline (300 in each arm). Australian residents were eligible for the trial if aged 18 to 75 years, reported having T2D diagnosed by a health professional, screened positive for depressive symptoms, and had access to an internet-enabled device. Exclusion criteria were as follows: inability to read English; presence of severe depressive symptoms; probable psychosis; high suicide risk; current participation in face-to-face psychotherapy for depression; recent (<2 months) change to antidepressant medication; and previous use of myCompass.

#### Stage I: Agreement in Principle to Participate

Practice recruitment took place in Victoria and New South Wales between September 2015 and February 2016. We conducted a multipronged approach to recruiting primary care professionals and their practices, including the following:

1. Emailing members of relevant professional organizations (eg, Australian Association of Practice Managers and Australian Diabetes Educators Association) and primary care providers subscribed to the Black Dog Institute’s eMH in practice training suite.
2. Promoting SpringboarD at relevant conferences (eg, Australian Primary Health Care Nurses Association).
3. Directly contacting 281 general practices with existing links to the University of New South Wales or the University of Melbourne via mail or phone.

Interested practices were offered a visit from a member of the research team (SF [Victoria] or JC [NSW]) at a time convenient to them to discuss the trial in more detail. Practices were advised that by consenting to take part in SpringboarD they also agreed to (1) invite patients with T2D to visit the trial website, by informing clients verbally and providing an information brochure during consultations, as well as by conducting a mailout to all patients of the practice aged 18 to 75 years with T2D (materials and postage provided) and (2) nominate a member of their team as the point of contact for the study (typically the practice manager or diabetes educator). In return, practices were offered Aus $50 per enrolled participant as recompense for time and effort and a report at the end of the project providing aggregated, deidentified data comparing their patients with those from other practices.

### Recruitment

#### General Practice Recruitment

As described by Bower et al [17], recruiting to primary care research can be considered across 4 stages. This paper has focused on the first 3 of these, in which (1) practices or primary care providers are initially approached and consent to take part in the research and (2) subsequently invite their patients who (3) then provide agreement to take part.

#### Stage II: Agreement to Recruit Patients

**Figure 1:**
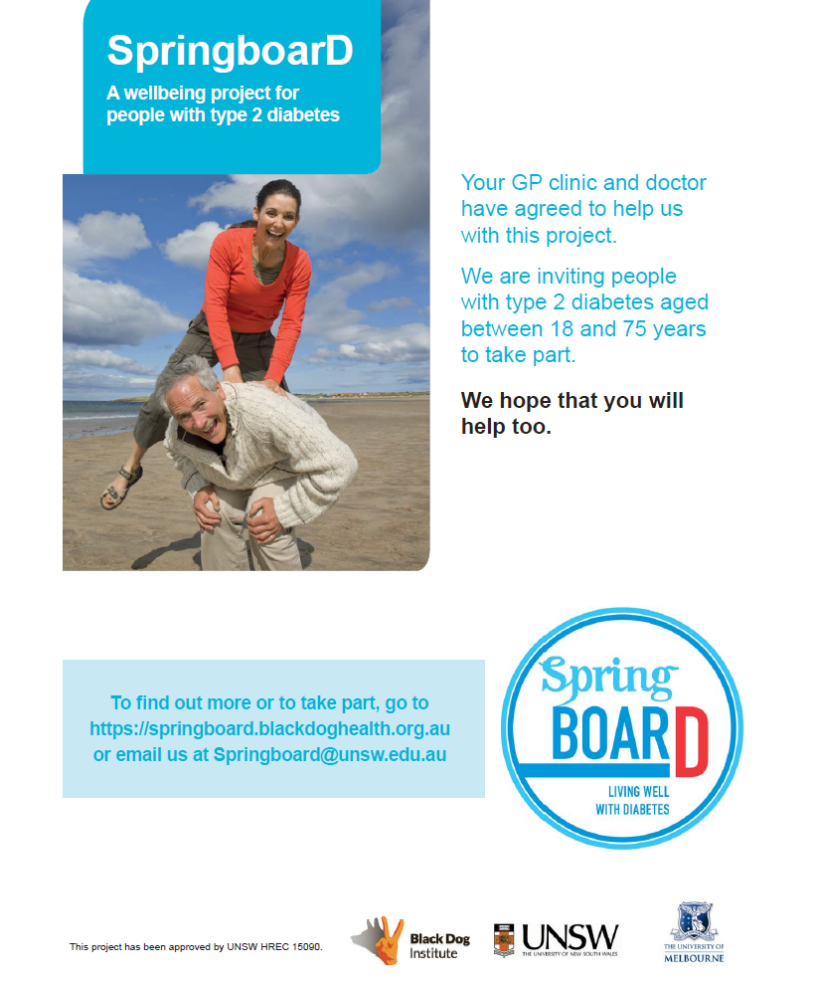
Poster for display in general practitioners (GPs) waiting rooms.

The nominated SpringboarD coordinators at each practice searched the practice database for patients with T2D aged between 18 and 75 years. Through this process, a total of 4569 patients were identified (on average, 169.2 per practice). Between November 2015 and March 2016, each of these patients was mailed a letter from the practice (using the practice letterhead), introducing the study and encouraging the patient to consider participating by visiting the trial website. Also enclosed was a letter of invitation from the SpringboarD research team, again providing the Web address and information about the option of entering a prize draw (Aus $100 grocery shopping voucher or tablet device) at each study time point, as recompense for the time and effort involved in study participation. All participating practices were provided with professionally produced posters (see Figure 1) and brochures to display in the waiting and consulting rooms.

Throughout 2016, recruitment proceeded slowly. We maintained regular contact including joint teleconferences with practice staff to identify and address barriers to patient recruitment. These discussions led to a series of practice engagement initiatives and reinforcing activities including the following:

1. The development of a recruitment Web portal for use during consultations where general practitioners (GPs) and diabetes educators could enter the patient’s name and email address to generate an automated email to the patient from the SpringboarD website with a link to the study page.
2. Development of a simple 3-step guide to introduce the study to patients using the Web portal above and laminated tags with the portal’s Web address that could be stuck on desktop computers as a prompt to navigate to it.
3. Provision of materials to remind practitioners about the trial, including trial-branded pens and magnets.
4. Offering alternative ways for the practice to promote the study, including providing text to upload on their website, or include in-patient newsletters, follow-up letters, or diabetes cycle of care reminders (including texts, emails, and phone calls) or having a research assistant located in the practice to invite patients to the trial as they presented for consultations.
5. World Diabetes Day (WDD) packs for patients, with WDD information, SpringboarD brochure, and a small gift (herbal tea and balloons), and suggestions about other ways the practice could link Springboard with their WDD activities.
6. A webinar for practice nurses to share experiences of introducing SpringboarD to patients and hear from a guest speaker (diabetes expert and practicing academic GP) about the latest developments in diabetes care and how these align with the goals of SpringboarD (ie, greater focus on impact and management of mental health comorbidities).
7. Scripts for practice staff to use in discussing the trial with potential candidates.

**Figure 2:**
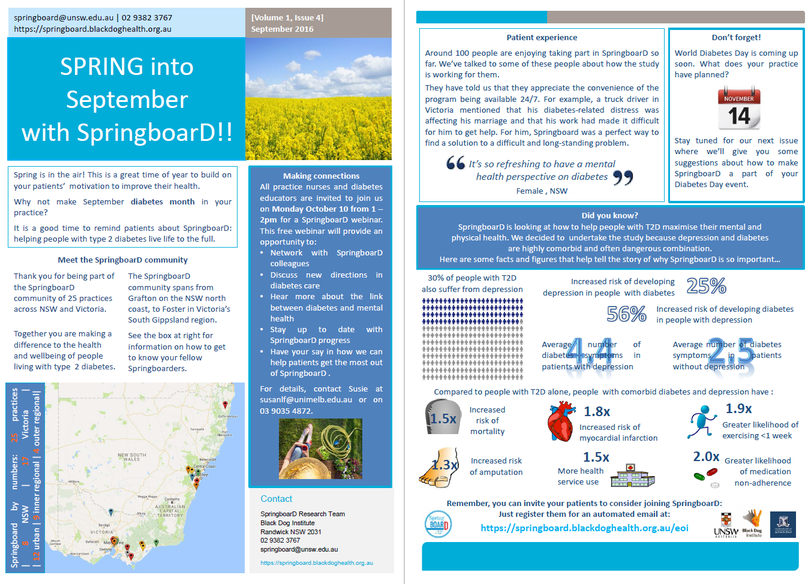
Example of newsletter sent to SpringboarD practices.

We also sent regular newsletters in both hard and soft copy to the nominated SpringboarD contact in each practice. These newsletters (see example in Figure 2) covered topics such as the rationale for and importance of study, provided reminders on using the Web portal, reiterated the offers of support in promoting the study to patients, introduced some of the SpringboarD practices and their staff (including a question and answer session about the benefits they perceived from taking part in the trial), and provided testimonials from participants.

#### Online Recruitment

Our online recruitment activities were mostly Facebook advertising campaigns that commenced in November 2015 and intensified from mid-2016 as it became clear that our efforts to recruit through general practice would not achieve the required sample size. We selected Facebook as it is the most commonly used social media platform in Australia, with around 60% of the population holding an account (14 million users in November 2015) and half of all Australians accessing Facebook at least once a day [18]. Recognizing the possible risks associated with Facebook advertising for potentially sensitive topics such as mental health [19], our recruitment approach included the following:

1. The use of a static advertisement that appeared on the right-hand side of the screen for people in the target demographic (located in Australia, aged between 18 and 65 or older, interested in T2D awareness or type 2 diabetes mellitus awareness).
2. Posting links to the study on the Black Dog Institute Facebook page and asking other organizations (eg, Diabetes Australia) to do the same.

**Figure 3:**
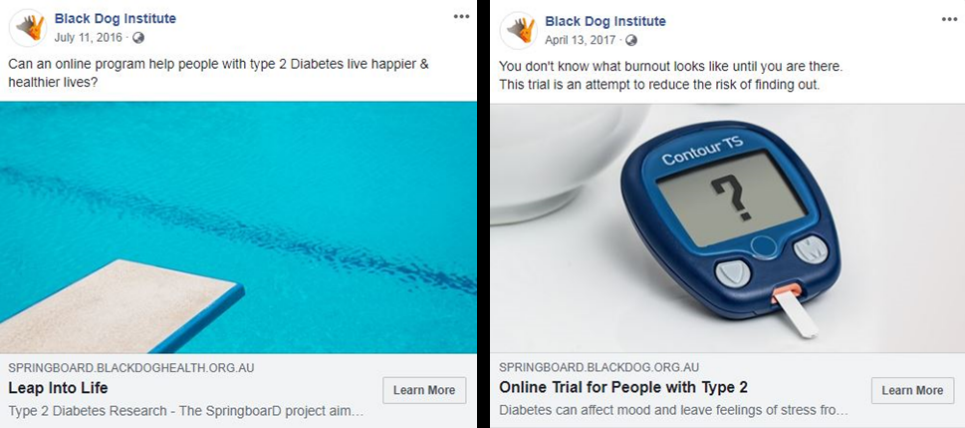
Examples of Facebook advertisements (Left: campaign 2; Right: campaign 4).

Over the course of the study, we ran 10 Facebook advertising campaigns, each building on the learnings of the last (Table 1). Through this iterative process, we identified the features that were most successful in encouraging link clicks and developed our advertisements accordingly (see examples in Figure 3). This resulted in an average click-through rate (ie, number of link clicks/number of unique users who had a SpringboarD advertisement appear on the screen) of 6.92% for campaigns 4 to 7, which compares favorably with the average click-through rate of 0.90% for Facebook advertisements generally and 0.83% for those that are health-related [20].

In addition to Facebook, we also advertised SpringboarD on the Black Dog Institute website and asked relevant organizations to do the same.

#### Other Recruitment Strategies

Our final approach toward recruitment involved specifically targeting populations in which we expected to have a high likelihood of recruitment success, *warm* populations with known T2D and an interest in research or diabetes care. This included sending email invitations and promoting the study at public diabetes forums and exhibitions, recruiting through diabetes organizations, word of mouth, and contacting members of 2 existing research cohorts (Table 2). The *diamond* cohort [21], recruited in 2005 through 30 general practices across Victoria, comprised 789 patients with depressive symptoms. Of these, 75 had reported having diabetes and consented to be contacted about relevant research. The 45 and Up study commenced in New South Wales in 2006 and recruited over 250,000 people aged over 45 years [22]. In total, 13,245 of these were identified as being potentially eligible for SpringboarD, of whom 4175 were randomly selected to be contacted by the Sax Institute on behalf of the SpringboarD team.

**Table 1:** Overview of Facebook recruitment campaigns.

Campaign number	Dates	Target audience	Budget (Aus$)	Reach^a^	Clicks	Click-through rate^b^ (%)	Comments
1	13 November-11 December 2015	Location: New South Wales, Victoria; Interests: T2D^c^ awareness or type 2 diabetes mellitus awareness	$420	23,047	1034	4.49	—^d^
2	4 April-30 June 2016	—	$500	9030	529	5.86	Small campaign (Aus $500 over 3 months) provided room to better define a target audience.
3	10-24 July 2016	Location: Australia; Interests: Type 2 diabetes mellitus awareness, T2D awareness, or diabetes awareness	$96	6510	254	3.90	Pilot testing new target audience.
4	1 August-30 September 2016	As per campaign 3	$904	29,912	2674	8.94	Increased budget and longer time frame allowed for monitoring and better analysis of which advertisements were performing the best during the campaign. This allowed us to pause posts with lower click-through rates and increased budget to higher performing advertisements, resulting in a proportional increase in daily click-throughs over time (from <20 per day early in the campaign to >100 in the second half).
5	23 October-22 November 2016	As per campaign 3	$500	20,764	1761	8.40	Nothing was changed from the previous campaign except that only the best performing posts were used. This infers that a working formula for recruitment for SpringboarD on Facebook had been achieved by this stage; continued through subsequent campaigns.
6a	12-30 April 2017	As per campaign 3	$440	39,868	2453	6.15	—
6b	12-19 April 2017	Location: High population areas of T2D in Sydney	$60	2233	103	4.61	Ceased owing to slow performance (ie, lower click-through rate) compared with concurrent campaign 6a
7	8 May-30 June 2017	As per campaign 3	$1200	87,981	5727	6.51	—
8	12-31 July 2017	As per campaign 3	$982	74,724	3157	3.66	—
9	1 August-30 August 2017	As per campaign 3	$4463	222,495	8314	3.28	—
10	1 September-30 September 2017	As per campaign 3	$5999	59,442	3486	4.98	—

^a^Reach: the number of unique users who had a SpringboarD advertisement appear on their screen.

^b^Click-through rate: link clicks/reach.

^c^T2D: type 2 diabetes.

^d^No additional comments provided.

**Table 2:** Recruitment campaigns through existing registries.

Registry name	Number of invitations sent	Date
Black Dog Institute Volunteer Research Registry	2378	November 2016
*diamond*^a^ cohort	75	May 2017
45 and Up cohort	4175	June 2017-September 2017
Members of diabetes organizations	25,550	April-June 2016

^a^Study name italicized as per [21].

### Data Collection

The data presented here were collected through the SpringboarD website as part of study enrollment procedures. Demographic information included age, gender, highest level of education, relationship status, and current employment.

#### Clinical Characteristics

Regardless of the referral source, all participants completed the same baseline questionnaire that assessed mental health and diabetes management. A series of forced choice items (yes/no) asked whether participants had ever sought professional help for their emotional health, had done so in the past 6 weeks, had ever been diagnosed with a mental illness, were currently taking medication for mental illness, had visited a GP in the past 6 weeks for diabetes, had been in hospital in the past 6 weeks for diabetes, or had been referred for a blood test (for glycated hemoglobin [HbA_1c_]) in the past 3 months. Participants were also asked to report the results of their most recent HbA_1c_ blood test, if known.

A number of validated measures in the screening and baseline questionnaires assessed mental health symptom severity and functioning. These included the Patient Health Questionnaire 9-item version (PHQ-9) [23] that assesses depressive symptom severity; the Generalized Anxiety Disorder 7-item scale (GAD-7) [24], a measure of anxiety symptom severity; the Work and Social Adjustment Scale (WSAS) [25] to assess the impact of current symptoms on functioning; and the Diabetes Distress Scale (DDS) [26] to assess distress attributed to diabetes. In all 4 measures, higher scores indicate poorer health and functioning.

#### Analysis

All analyses were conducted using SPSS version 24 [27]. Information on the recruitment source was collapsed into 3 categories: (1) general practice; (2) online (social media, search, and link from another website); and (3) other (research registries, diabetes forums and exhibitions, diabetes organizations, and word of mouth). We grouped word of mouth together with research registries and diabetes organizations as we considered all 3 to be reaching potentially *warm* populations, likely to have a particular interest in the research.

Average scores and prevalence of participant characteristics were compared across the 3 groups using analysis of variance (ANOVA) for continuous data, with effect sizes calculated using eta squared (η²). Preliminary analyses revealed that variance in age was nonhomogenous across groups, and we therefore employed Welch’s ANOVA for this variable [28], with post hoc differences examined using the Games-Howell test. For all other continuous variables, differences between groups were compared using standard ANOVA and post hoc differences were examined using Tukey HSD (which, in SPSS, automatically implements the Tukey-Kramer modification to account for unequal group sizes). For categorical data, we employed Fisher exact test owing to its robustness to small sample sizes; where overall results indicated a significant difference, pairwise z-tests were conducted to identify where differences lay. Effect sizes for categorical analyses are reported using Cramer V. For all analyses, Bonferroni corrections were applied for multiple testing, and missing data were handled using a pairwise deletion strategy to maximize the number of participants retained.

## Results

**Figure 4:**
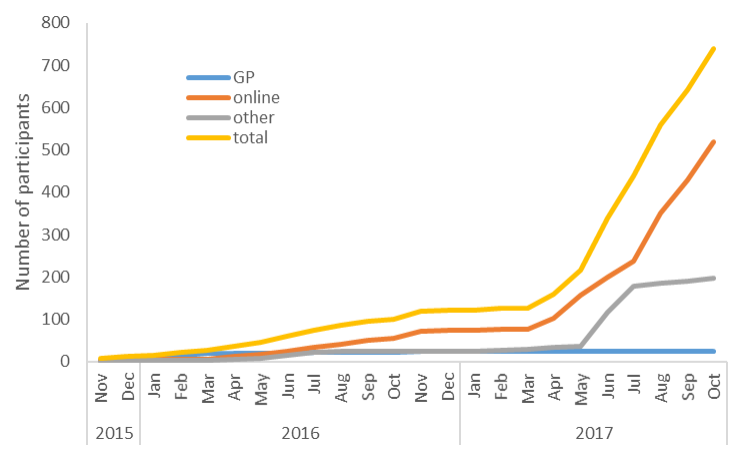
Cumulative enrolment in SpringboarD by recruitment source. GP: general physician.

Between November 2015 and October 2017, a total of 888 people met the eligibility criteria. Of these, 780 completed the baseline questionnaire and enrolled in SpringboarD. In total, 40 participants did not provide information on the recruitment source and were excluded from further analysis; hence, the sample reported here comprises 740 participants. Figure 4 provides an overview of the recruitment of these participants over time.

### Outcomes of General Practice Recruitment

In total, our general practice recruitment efforts resulted in 27 practices consenting to take part in the trial. Despite our intensive approach to engaging these sites in patient recruitment, only 24 trial participants indicated they had learned about SpringboarD through their GP (0.53% of those who were sent an invitation letter from their GP). Most enrollments occurred soon after the initial invitation letter was mailed, and only 3 GP enrollments were recorded after April 2016.

Interviews with nominated SpringboarD coordinators identified several barriers to inviting patients to the trial, including the following:

1. Perception by some practice staff of a mismatch between trial participation requirements and the diabetes population. Some practice staff reported that because many of their patients with diabetes were older and therefore possibly less computer literate, they chose not to mention the study to them. In rural areas, some of our coordinators considered low socioeconomic status and internet or mobile phone access to be additional barriers to participation in the trial.
2. Practice nurses reported that because acute health issues must take priority in consultations, they often felt it impractical or inappropriate to bring up research (even when it was relevant to the patient’s presentation).
3. Cycle of care reminders were not used in some practices, meaning that the SpringboarD prompts offered by researchers to include in text, mail, or phone contacts were not taken up.
4. Some SpringboarD coordinators reported that patients had previously been unhappy with the practice sending third party emails, which made several practices reluctant to use the Web portal to send automated emails despite this being done in a GP or practice nurse consultation, with patient consent.
5. Community or practice events occasionally made it difficult for staff to focus on SpringboarD. This included routine events, such as staff leave, and unexpected events, for example, a dairy crisis in a region of Victoria affecting the entire community, including the local practice’s day-to-day operations.

### Outcomes of Online Recruitment

Online recruitment was relatively slow through our early campaigns, as we tested different target audiences and nomenclature. In campaign 4, we identified that promoting the study as one trying to reduce *burnout related to diabetes* attracted most Facebook users and continued this approach through subsequent campaigns. By the end of recruitment, Facebook accounted for the majority of participants (462/740; 62.4%). Also included in the group of participants who found out about SpringboarD online were 58 individuals (7.8% of the total sample [58/740]) who came to the trial through an internet search or link from another website.

### Outcomes of Other Recruitment Strategies

Recruitment through other sources yielded just over a quarter of the sample overall (196/740; 26.5%), with marked variation in effectiveness and efficiency. Sending invitations to members of existing research registries was most successful, accounting for 75.5% of participants in the *other* recruitment category (148/196; 20.0% of the overall sample). On the contrary, word of mouth generated very few participants (12/740; 1.6%).

Table 3 shows the number of participants indicating each recruitment source and the estimated number of people reached by each recruitment campaign. Note that higher numbers of recruited participants are associated with a larger population of people who received a study invitation or were targeted by an online advertisement. Overall, the conversion rate from potential exposure to promotional materials to trial enrollment was less than 1%, ranging from a low of 0.09% (520/573,333) from online recruitment and 0.61% (196/32,178) from other sources. These figures should be interpreted in light of the fact that online figures represent the total reach across multiple Facebook campaigns and individuals may be counted more than once. However, other potential denominators (such as the number of link clicks) do not translate across the recruitment source, and thus, we considered the total reach of each recruitment strategy to be the most appropriate point of comparison.

**Table 3:** Number of participants recruited by source.

Recruitment source (general and specific)		Participants recruited^a^, n (%)	Estimated reach^b^	Participants as percentage of estimated reach
**Online**		**520 (70.3)**	**573,733**	**0.09**
	Facebook	462 (62.4)	573,733^c^	0.08
	Internet search/link from other website	58 (7.8)	—^c^	—
**Other**		**196 (26.5)**	**32,128**	**0.61**
	Research registries	148 (20.0)	6628	2.23
	Diabetes organization	36 (4.9)	25,550	0.14
	Word of mouth	12 (1.6)	—	—
General practice		24 (3.2)	4569	0.53
Total		740 (100.0)	610,480	0.12

^a^Excludes 40 participants who did not indicate a recruitment source.

^b^Note that individuals may be exposed to multiple recruitment campaigns, both within and across sources.

^c^Unknown.

### Characteristics of Participants by Recruitment Source

A comparison of participant characteristics at baseline suggested that our 3 different approaches to participant recruitment yielded groups that were broadly similar. Overall differences were identified on only 5 of the 15 variables considered, including 3 demographic and 2 clinical characteristics, as described below.

In terms of demographic variables, overall group differences were evident in gender, age, and employment. Pairwise testing revealed that a significantly lower proportion of participants recruited online were male compared with both the GP and other groups that did not differ from each other (Table 4). Looking at age, post hoc testing indicated that the overall significant but weak effect was because of online participants being significantly younger than those recruited through either general practice or other sources (Table 5). Potentially as an artifact of this age difference, participants recruited online were also more likely to be employed than those recruited through other sources, with no difference in employment rates between the online and GP groups or the GP and other groups. There was also a nonsignificant trend toward educational differences between groups, driven by a tendency for participants recruited online to be less likely to have a university degree than those recruited through other sources.

**Table 4:** Baseline characteristics of participants recruited through general practice, online, and other sources (categorical).

Characteristics		General practice (N=24), n (%)	Online (N=520), n (%)	Other (N=196), n (%)	*P* value	Effect size (Cramer V)
Gender (male)		16 (66)	141 (27.1)	92 (46.9)	.00^a,b^	0.224
**Relationship status**					**.23**	**0.070**
	Married/de facto	16 (66)	332 (63.8)	139 (70.9)	—^c^	—
	Separated/divorced	4 (16)	85 (16.3)	30 (15.3)	—	—
	Single	3 (12)	83 (16.0)	17 (8.7)	—	—
	Widowed	1 (4)	20 (3.8)	10 (5.1)	—	—
	Employed (vs not)	9 (37)	283 (54.4)	67 (34.2)	<.001^b^	0.182
**Highest level of education**					**.006**	**0.108**
	High school	10 (41)	170 (32.7)	44 (22.4)	—	—
	University	8 (33)	146 (28.1)	82 (41.8)	—	—
	Apprentice/trade	2 (8)	34 (6.5)	15 (7.7)	—	—
	Diploma/other	4 (16)	170 (32.7)	55 (28.1)	—	—
**Mental health care**						
	Sought professional help for emotional health in the past 6 weeks	4 (16)	77 (14.8)	33 (16.8)	.78	0.025
	Current medication for mental illness	5 (20)	178 (34.2)	60 (30.6)	.29	0.060
**Diabetes care**						
	Visited GP^d^ in the past 6 weeks for diabetes	14 (58)	299 (57.5)	120 (61.2)	.66	0.033
	Been to the hospital in the past 6 weeks for diabetes	1 (4)	23 (4.4)	6 (3.1)	.65	0.030
	Had HbA_1c_^e^ test in the past 3 months	18 (75)	394 (75.8)	164 (83.7)	.04	0.075

^a^Significant difference between general practice and online group (Bonferroni-corrected pairwise comparison).

^b^Significant difference between online and other group (Bonferroni-corrected pairwise comparison).

^c^Posthoc analysis not conducted due to nonsignificant main effect.

^d^GP: general practice.

^e^HbA_1c_: glycated hemoglobin.

Looking at clinical characteristics, no differences between groups were found in any variables related to mental health care or diabetes management (Table 4). Participants recruited online reported higher PHQ-9 and DDS scores (Table 5) and showed a trend toward higher GAD-7 scores than those in the *other* group, although these differences should be interpreted in light of several caveats. First, in all cases, the effect size was small. Second, a cutoff of 10 is typically used on the PHQ-9 to delineate clinically significant depressive symptoms [23] and both the online and other groups were just above this cutoff, whereas the GP group was below it. Finally, all groups fell below the cutoff of 3 on the DDS, indicating mild distress not requiring clinical attention. Therefore, differences between the online and other groups in these measures may be statistically but not clinically significant.

**Table 5:** Baseline characteristics of participants recruited through general practice, online, and other sources (continuous).

Characteristics	General practice (N=24), mean (SD)	Online (n=520), mean (SD)	Other (n=196), mean (SD)	Significance		Effect size (eta-squared [η²])
				*F* test value (*df*)	*P* value	
Age	62.99 (8.58)	55.32 (10.41)	63.07 (7.90)	59.49 (2, 734)	<.001^a,b^	0.012
PHQ-9^c^	8.71 (4.51)	11.51 (3.94)	10.08 (4.02)	13.46 (2, 737)	<.001^b^	0.035
GAD-7^d^	6.33 (4.78)	7.79 (4.19)	6.68 (9.98)	5.97 (2, 737)	.003	0.016
WSAS^e^	10.42 (7.28)	13.29 (7.68)	12.79 (8.82)	1.64 (2, 737)	.20	0.004
DDS^f^	2.03 (0.85)	2.71 (0.95)	2.24 (0.88)	22.04 (2, 737)	<.001^b^	0.056
HbA_1c_^g^	7.00 (1.04)	8.11 (5.82)	7.31 (4.18)	0.76 (2, 289)	.469	0.005

^a^Significant difference between general practice and online group (Bonferroni-corrected pairwise comparison).

^b^Significant difference between online and other group (Bonferroni-corrected pairwise comparison).

^c^PHQ-9: Patient Health Questionnaire 9-item.

^d^GAD-7: Generalized Anxiety Disorder 7-item.

^e^WSAS: Work and Social Adjustment Scale.

^f^DDS: Diabetes Distress Scale.

^g^HbA_1c_: glycated hemoglobin.

## Discussion

Despite considerable time, labor, and financial investment, as well as employing many recruitment strategies previously reported to improve the likelihood of success, we were unable to recruit successfully through general practice to a trial of a Web-based mental health intervention for people with T2D and mild-to-moderate depression. The characteristics of the small group of participants who were recruited to the trial via general practice were broadly comparable with the larger sample, suggesting that difficulties in primary care recruitment are not a significant threat to internal validity. However, as is common in research, across all recruitment sources, only a small proportion of people who were exposed to SpringboarD recruitment materials accepted the invitation to take part—and thus the broader challenge of research generalizability remains.

### Comparison With Previous Research

Our findings that online recruitment yielded the greatest number of participants are consistent with some previous research showing this approach to be more time- and/or cost-effective than more traditional methods of RCT recruitment [10,29]. However, other authors have reported comparable or even lower efficiency of online recruitment compared with other sources [9,30,31]. Similarly, the evidence as to how online recruitment affects sample characteristics remains limited and equivocal. Published reviews comparing the characteristics of participants recruited via social media and other sources found no consistent trend in the effect of recruitment source on age, with social media variously associated with younger, older, or same aged samples [31,32]. More consistent was the tendency to find no difference in gender, level of education, and relevant clinical characteristics, although exceptions in both directions have also been identified [30-36]. Thus, our finding that participants recruited online are slightly more likely to be younger, female, employed, and more distressed supports some, but not all, previous reports. The jury appears to still be out when it comes to identifying meaningful patterns in the effect of recruitment source and sample characteristics. It is worth noting that much of our understanding of this relationship relates to cohort studies rather than RCTs and to studies of smoking cessation more so than other health behaviors [30,31]. This study therefore builds the evidence related to randomized trial recruitment while extending previous research to a new population (people with depressive symptoms and diabetes).

### The Challenge of General Practice Recruitment: There Is No Right Way

General practice recruitment proved difficult despite an intensive effort, including involvement of GPs early in the trial design. This may reflect the fact that eMH has not yet been normalized into everyday practice, despite significant investment in raising GP awareness of these interventions (including nation-wide training for GPs—eMHPrac—funded by the Australian Government Department of Health and development of guidelines on when, how, and why to refer patients to these programs [37]). Alternatively, efforts to engage GPs in eMH may not have translated to engaging other key primary health professionals, such as diabetes educators. The SpringboarD trial was not designed to explore attitudes toward eMH among GPs and other primary care providers, and as far as we are aware, this topic has not been investigated previously. It therefore represents an important avenue for future enquiry.

Of course, our limited success in recruiting through general practice may not have been only due to barriers at the provider level, with patient response to the initial mailout also limited. This echoes our experience with people with type I diabetes, suggesting that diabetes itself may pose a barrier to research participation for some people. Participants in a study by Clarke et al [38] expressed limited awareness of the link between mental health and diabetes and spoke about the *double stigma* of chronic mental and physical comorbidity. This suggests that providing further education around the bidirectional relationship between mental and physical health may have benefits in terms of both patient outcomes and the future of primary care research for people with T2D.

There is limited evidence for how to optimize recruitment in primary care, but the general agreement is that a multifaceted approach is best [7]. However, identifying the most successful strategies is more complicated than it might appear. As noted by Graffy et al [39], both trials that successfully recruit, and those that do not, often report using many of the same recruitment strategies. Our SpringboarD experience certainly attests to this. Our first approach to recruitment through general practice was to send invitation letters to all patients with T2D aged between 18 and 75 years. We have previously used this approach with success in both randomized trials [40] and cohort studies [21], where 28.57% (5742/20,100) and 43.12% (7667/17,780) of patients, respectively, responded to a mailed invitation letter. Similarly, our recruitment approach shared many similarities with Reed et al’s [5] successful recruitment of older adults to a trial of chronic disease self-management, where 38.12% (634/1663) of patients who were mailed a letter of invitation expressed interest in participating in the trial. In the current trial, however, the number of participants who indicated they found out about SpringboarD through their general practice was less than 1% of the number of invitations mailed.

The general consensus is that primary care recruitment is most successful when patient eligibility criteria are simple and practice staff are not expected to spend time managing patient consent [6,7]. We designed our recruitment approach with this in mind and asked practice staff only to invite patients with T2D to visit the study website; all eligibility screening was completed online. To further reduce the burden on practice staff, we offered practices the option of having a research assistant located in the practice to invite patients directly, an approach we have previously found effective [41]. Our experience suggests that although this approach can help to achieve sample size targets, practices can feel uncomfortable with a shift to researcher-led recruitment midtrial if this is not initially agreed to.

### Who Takes Part in an e-Mental Health Trial?

SpringboarD participants from all recruitment sources were generally similar to each other. This is perhaps not surprising when we consider that the majority of Australians can be categorized as both GP patients and Facebook users; the two are far from mutually exclusive and it is possible that internet searchers were looking for SpringboarD after hearing about it through our active recruitment strategies. Just as eMH interventions are designed to supplement traditional mental health care, participants recruited online should not be considered to exist outside the health care system. Over 80% of Australians visit a GP once per year, and this number rises to 95% among people with a long-term health condition [42]. Thus, Facebook users with T2D may be considered a subset of the general practice population, who happen to enter trials such as SpringboarD via a different path.

One key difference between our recruitment groups was that a greater proportion of the participants recruited online were female. This gender difference is consistent with findings that women are more likely to engage in help-seeking behaviors, generally, and searching for health information online, specifically [43]; they may have been more likely to find and follow the SpringboarD invitation link. The population prevalence of diabetes is similar in men and women overall (6% and 5%, respectively), but among older age groups (55 and older), the condition is more common in men [44]. One of the often-cited benefits of online recruitment is that it can reach populations that are traditionally hard to engage. When it comes to mental health research, men are one such population, but in this study, online recruitment did little to overcome this gap. The significance of the potential to reach men through GP or other avenues of recruitment should not be dismissed. Particularly where the research question is especially important to men’s health, the investment in time and resources required to recruit through primary care may be justified, if, of course, recruitment targets can be achieved within the study timeline and funding.

That different recruitment pathways yielded mostly similar groups provides reassurance that the effect of recruitment source on sample characteristics is negligible. However, questions about selection effects remain. The individuals who enrolled in SpringboarD and are reported here represent less than 1% of people who were potentially exposed to one or more invitations or advertisements. Our findings support those of previous authors, who reported similar biases in both online and offline recruitment methods [36], and similar characteristics of participants who enroll in trials of eMH and traditional psychological and pharmacological treatments [45]. In other words, people who take part in mental health research differ from people who do not, regardless of the recruitment approach or intervention being tested. Thus, although recruiting online may be a solution to the (significant) challenge of obtaining an adequate sample size, it does not address the broader issue of overcoming selection bias in community-based recruitment. As the saying goes, everything old is new again, and in the new era of Web-based research, we continue to face the old problem of representativeness.

### Limitations

The findings reported above should be considered in light of certain limitations. First, it should be noted that information on the recruitment source was only collected for patients who were eligible for the trial. In total, 6145 people visited the SpringboarD landing page and 2849 of those completed the screening questionnaire but were ineligible; whether these individuals were spread evenly across recruitment sources is unclear. In addition, we did not collect information from participating general practices about all patients who were mailed an invitation letter and it is therefore unclear how participants and nonparticipants compare. Also note that although we base our uptake calculations on the estimated *reach* of each campaign, we have no evidence that Facebook users actually saw the advertisement or that invitation letters reached or were opened by GP patients and research registry participants. Our calculations therefore potentially overestimate the number of potential participants and therefore underestimate the proportion of those who enrolled in the trial. It is also possible that this over or underestimate differs by recruitment source; potential participants may be more likely to open a letter from their GP than notice an advertisement on Facebook. Finally, this study relates to recruitment only; retention is a further significant challenge in all trials (particularly those conducted online). The interested reader is referred to Clarke et al [14] for further information on participant retention in the SpringboarD RCT.

### Conclusions

Consistent with previous research, this study showed that recruiting to an RCT through general practice was difficult and ultimately unsuccessful. Our findings support previous recommendations that working with practice staff to identify the best approach early on may improve the likelihood of success. Furthermore, ensuring a match between recruitment strategies and the nature of the trial or intervention being tested may be beneficial. Nonetheless, trial participants recruited via general practice were not markedly different from those recruited online or through other means. This suggests that regardless of how people came into the trial, recruitment materials attracted those with similar characteristics. Thus, the threat of different recruitment sources to internal validity appears minimal. GPs can be reassured that current evidence for the effectiveness of eMH interventions, frequently generated from samples recruited online, is likely similar to that obtained if the sample was obtained entirely through general practice.

## Abbreviations

ANOVA: analysis of variance
DDS: Diabetes Distress Scale
eMH: e-mental health
GAD: Generalized Anxiety Disorder
GPs: general practitioners
HbA _1c_: glycated hemoglobin
HREC: Human Research Ethics Committee
PHQ: Patient Health Questionnaire
RCT: randomized controlled trial
T2D: type 2 diabetes
WDD: World Diabetes Day
WSAS: Work and Social Adjustment Scale
